# Post-Transcriptional Regulation of Gene Expression in *Yersinia* Species

**DOI:** 10.3389/fcimb.2012.00129

**Published:** 2012-11-09

**Authors:** Chelsea A. Schiano, Wyndham W. Lathem

**Affiliations:** ^1^Department of Microbiology-Immunology, Northwestern University Feinberg School of MedicineChicago, IL, USA

**Keywords:** Ysr, sRNA, T3SS, Hfq, plague, yersiniosis, thermosensor, riboswitch

## Abstract

Proper regulation of gene expression is required by bacterial pathogens to respond to continually changing environmental conditions and the host response during the infectious process. While transcriptional regulation is perhaps the most well understood form of controlling gene expression, recent studies have demonstrated the importance of post-transcriptional mechanisms of gene regulation that allow for more refined management of the bacterial response to host conditions. *Yersinia* species of bacteria are known to use various forms of post-transcriptional regulation for control of many virulence-associated genes. These include regulation by *cis*- and *trans*-acting small non-coding RNAs, RNA-binding proteins, RNases, and thermoswitches. The effects of these and other regulatory mechanisms on *Yersinia* physiology can be profound and have been shown to influence type III secretion, motility, biofilm formation, host cell invasion, intracellular survival and replication, and more. In this review, we discuss these and other post-transcriptional mechanisms and their influence on virulence gene regulation, with a particular emphasis on how these processes influence the virulence of *Yersinia* in the host.

## Introduction

The three species of *Yersinia* pathogenic to humans encounter a variety of challenges throughout the course of their life cycles, including from the host immune system as well as from various environmental sources. Two of these species, *Yersinia enterocolitica* and *Y. pseudotuberculosis*, are gastrointestinal pathogens that are transmitted to mammals through the fecal-oral route from water and other natural reservoirs (Vantrappen et al., 1982; Rich et al., 1990). In these environmental niches they encounter a range of temperatures and pH as well as other potentially hostile microbes, to which *Y. pseudotuberculosis* and *Y. enterocolitica* must respond appropriately in order to survive and maintain homeostasis (Calvo et al., 1986; Pepe et al., 1994; Harrison et al., 2000; Palonen et al., 2010). Following ingestion by a mammalian host, the enteric *Yersiniae* must adapt to higher temperatures and pass through the acidic environment of the stomach before reaching the small intestine where invasion of the deeper tissue occurs (Miller and Falkow, 1988; Marra and Isberg, 1997; Nagel et al., 2001; Abdela et al., 2011). Upon invasion *Y. enterocolitica* and *Y. pseudotuberculosis* are confronted with host immune cells such as dendritic cells, macrophages, and neutrophils that the *Yersiniae* must resist in order to disseminate and propagate the infection (Simonet et al., 1985; Monack et al., 1998; Laws et al., 2011).

Similarly to its enteric ancestors, *Y. pestis*, the causative agent of plague, has two distinct phases of its life cycle (Perry and Fetherston, 1997; Gage and Kosoy, 2005). *Y. pestis* does not live freely in the soil or water, instead it typically colonizes fleas in order to be transmitted to mammals (Lorange et al., 2005), and maintains considerable genetic material dedicated to this part of its life cycle (Hinnebusch et al., 2002; Darby et al., 2005; Vadyvaloo et al., 2007; Sebbane et al., 2009; Chouikha and Hinnebusch, 2012). In addition, *Y. pestis* has been shown to colonize multiple mammalian organs, including the lymph nodes, spleen, lungs, and blood, and the infection of these distinct sites in the body results in the expression of different subsets of genes (Tieh and Landauer, 1948; Lathem et al., 2005; Sebbane et al., 2005; Lawson et al., 2006; Chauvaux et al., 2007).

The diversity and flux of conditions that *Yersiniae* must adapt to throughout their life cycles result in significant changes in metabolic, cell surface, and virulence factor gene expression, which are modulated through complex regulatory networks that allow the bacteria to respond appropriately and rapidly. These pathogenic *Yersiniae*, and bacteria in general, regulate gene transcription as a major means of controlling the cohort of factors expressed under any given condition. For instance, sigma factors, promoter sequences, and transcriptional activators and repressors all directly impact the frequency with which any particular gene is transcribed, and the contribution of transcriptional regulators to gene expression is directly linked to mechanisms that sense the environment and needs of the bacterium at any given moment (Cornelis et al., 1989, 1991; Iriarte et al., 1995a,b; Trulzsch et al., 2001; Nagel et al., 2003; Green and Darwin, 2004; Brzostek et al., 2007; Gao et al., 2008; Raczkowska et al., 2011). In addition, the production and use of bacterial virulence factors are energy intensive processes, and the *Yersiniae* must tightly control their synthesis in order to maximize survival, replication, and spread. For example, the Yop-Ysc type III secretion system (T3SS) of *Yersinia*, which is essential for mammalian virulence, requires a large energetic commitment from the bacteria for production and effector protein translocation, and inappropriate expression or mis-targeting of the system can have significant impact on the success of the bacteria in the host (Woestyn et al., 1994; Cornelis et al., 1998; Aepfelbacher and Heesemann, 2001; Blaylock et al., 2006; Shao, 2008). However, when the T3SS is deployed upon contact with host immune cells, the system prevents phagocytosis and activation of an effective immune response against the bacteria (Cornelis and Wolf-Watz, 1997; Shao, 2008). It is no surprise, then, that dozens of studies have revealed an intricate and multi-layered regulatory network that governs the expression and synthesis of the T3SS. Many of these studies have focused on the role of transcriptional regulation of this system; in recent years, however, there have been a number of reports that have elucidated post-transcriptional and post-translational mechanisms of regulation as well.

Post-transcriptional regulation is a key step in the control of bacterial gene expression, and our understanding of the mechanisms involved therein is rapidly growing. The regulatory elements involved in post-transcriptional regulation encompass any interaction of molecules with mRNA transcripts that affect translation of the message into protein products. This review focuses on the mechanisms that are specific to this step (as distinct from post-translational regulation, which occurs once protein has been synthesized). The post-transcriptional regulatory mechanisms of the *Yersiniae* include the use of RNA-binding proteins, small regulatory RNAs, other non-coding RNAs, thermosensors, RNases, and others. Post-transcriptional regulation provides a powerful way for the bacteria to more rapidly adjust to the changing environment during the *Yersinia* life cycle and to fine tune gene expression to the needs of the cell. Indeed, this is because translation can occur more quickly from existing transcripts rather than requiring *de novo* transcription. In this review we discuss specific examples of post-transcriptional regulation in *Yersinia* that may be involved in pathogenesis or other aspects of *Yersinia* physiology (Table 1) and we provide a comparative context for similar and/or divergent mechanisms in other pathogenic bacteria.

**Table 1:** **Post-transcriptional regulators, targets, and functions in *Yersinia* spp**.

Name	Target	Function/mechanism	Reference
**RNA-BINDING PROTEINS**			
YopD	5′ UTR of *yopQ* in *Y. enterocolitica* (*yopK*); maybe others	Repression of transcription via ribosome competition or transcript degradation	Williams and Straley (1998), Chen and Anderson (2011)
LcrH	Same as YopD	Functions in complex with YopD	Anderson et al. (2002)
YscM1/YscM2 (LcrQ)	Not determined; predicted to be 5′ UTR of *yop* mRNAs	Negative regulation of T3SS; possibly same as YopD and/or with YopD	Cambronne and Schneewind (2002)
CsrA	GGA-motifs in the 5′ UTR; pleiotropic	Global carbon storage regulation; represses by ribosome competition or transcript degradation	Dubey et al. (2003), Baker et al. (2007), Heroven et al. (2008), Heroven et al. (2012)
SmpB	SsrA and A site of stalled ribosome	Ribosome rescue; molecular mimicry; enters into empty A site of a ribosome 1:1 ratio w/SsrA	Karzai et al. (1999), Okan et al. (2006), Okan et al. (2010), Neubauer et al. (2012)
Hfq	AU-rich regions of RNA; pleiotropic	sRNA chaperone; stabilizes interaction of sRNA w/mRNA	Nakao et al. (1995), Moller et al. (2002), Geng et al. (2009), Schiano et al. (2010)
**NON-CODING RNAs**			
SsrA	Stalled ribosomes	Ribosome rescue; tRNA and mRNA Replaces incomplete transcript in ribosome; allows termination; tags for degradation	Karzai et al. (2000), Okan et al. (2006)
CsrB/CsrC	CsrA	Highly structured RNAs sequester CsrA w/multiple GGA-motifs	Liu et al. (1997), Romeo (1998), Heroven et al. (2012)
SgrS/Ysr150	5′ UTR of *ptsG*	Inhibits *ptsG* translation to stop the synthesis of new glucose transporters	Wadler and Vanderpool (2007), Horler and Vanderpool (2009), Wadler and Vanderpool (2009), Rice and Vanderpool (2011)
RybB/Ysr48	5′ UTR of many *omp* transcripts, incl. *ompA*, *ompC*, *ompD*, *ompF*, *ompW*	Regulates outer membrane protein composition; promotes accelerated mRNA degradation	Vogel and Papenfort (2006)
MicF	5′ UTR of *ompF*	Same as RybB	Andersen et al. (1989), Schmidt et al. (1995), Delihas and Forst (2001), Delihas (2003), Vogel and Papenfort (2006)
MicA/Ysr7	5′ UTR of *ompA*	Same as RybB	Udekwu et al. (2005), Vogel and Papenfort (2006)
OmrA/Ysr149	5′ UTR of *ompT*	Same as RybB	Guillier and Gottesman (2006), Vogel and Papenfort (2006)
GlmY/Ysr147	*glmS* transcript	Stabilizes *glmS* mRNA; positive regulation of cell wall synthesis	Kalamorz et al. (2007), Urban et al. (2007), Reichenbach et al. (2008), Gopel et al. (2011)
GlmZ/Ysr148	GlmY – unknown if direct or indirect	Regulates amount of GlmY	Kalamorz et al. (2007), Urban et al. (2007), Reichenbach et al. (2008), Gopel et al. (2011)
GcvB/Ysr45	*dppA* transcript	Repression of periplasmic-binding protein component of the dipeptide transport system	McArthur et al. (2006), Pulvermacher et al. (2008, 2009)
YenS	*yenI* transcript; potential secondary targets	Positive regulation of motility; inhibits translation and promotes degradation of *yenI* mRNA	Tsai and Winans (2011)
RyhB/Ysr48	*sdhCDAB* operon transcript, others	Fur-repressed sRNAs; negative post-transcriptional regulation of targets	Masse and Gottesman (2002), Vecerek et al. (2003), Deng et al. (2012)
SraG	YPK_1206-05 operon transcript	Direct regulation; unknown function	Lu et al. (2012)
Ysr29	*ureC*, *ahpC*, *gst*, *frr*, *rpsA*, *ompA*, *groEL*, *dnaK*	Hfq-dependent; negative regulation and positive regulation of different targets	Koo et al. (2011)
Ysr35	Unknown	Role in virulence of *Y. pestis* and *Y. pseudotuberculosis*	Koo et al. (2011)
Antisense to *pla*	*pla*	Unknown; predicted to repress translation of Pla	Sodeinde and Goguen (1989)
Yp-sR7	50 s Ribosomal protein (*rplK*)	Expressed in exponential phase; *cis*-encoded; unknown function	Qu et al. (2012)
Yp-sR3	YP_1329 and YP_1330	Expressed in stationary phase; *cis*-encoded across operon; unknown function. May regulate these putative membrane proteins	Qu et al. (2012)
Yp-sR8	Unknown	Expressed in stationary phase; unknown function	Qu et al. (2012)
**RNases**			
RNase E	Many; T3SS	Inhibits export/secretion of T3SS effector proteins	Yang et al. (2008)
PNPase	Many; T3SS	Same as RNase E	Rosenzweig et al. (2005), Rosenzweig et al. (2007), Rosenzweig and Schesser (2007)
**THERMOSENSORS**			
*yscW-lcrF* intergenic region	*lcrF* (*virF*) transcript; *cis*-acting RNA	Two-stem loop structure restricts access of ribosome to SD sequence at 25°C; but not at 37°C. Proper function required for virulence	Hoe and Goguen (1993), Bohme et al. (2012)
**RIBOSWITCHES**			
*mgtA*	5′ UTR of *mgtA*	High Mg^2+^ concentration leads to early Rho-dependent termination of *mgtA* transcription through conformational change in the RNA; regulates magnesium transporter production	Korth and Sigel (2012)

## Regulatory RNA-Binding Proteins

The ribosome is the primary RNA-binding protein in the cell and is required for the translation of mRNA into protein. However, there are other proteins that are known to bind to mRNA, and by doing so regulate translation initiation, mRNA stability, and half-life of the message (Anderson et al., 2002; Brennan and Link, 2007). These proteins often compete with the ribosome for binding to exert their regulatory effects (Anderson et al., 2002; Wang et al., 2005).

### T3SS regulators

**Figure 1:**
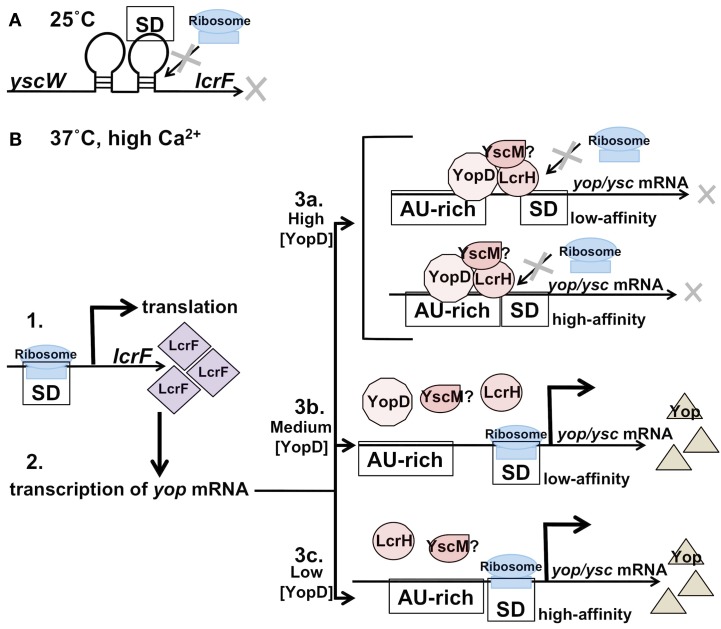
**Post-transcriptional regulation of the *Yersiniayop-ysc* T3SS: LcrF thermosensor and the YopD-LcrH complex**. **(A)** At 25°C the T3SS is repressed at the transcriptional level. The thermosensor in the UTR between the *yscW* and *lcrF* transcriptis in the closed conformation at 25°C, which prevents ribosome binding and translation initiation of *lcrF*. **(B)** 1. At 37°C the thermosensor of *lcrF* is in the open conformation, allowing translation, and production of LcrF to proceed. 2. LcrF activates transcription of *yop*/*ysc* genes. 3. Translation of a cohort of *yop*/*ysc* mRNAs is repressed by a YopD-LcrH (YscM) complex binding in the 5′ UTR until favorable translocation conditions exist. 3a. Under high YopD concentrations (little-to-no secretion exists) the YopD complex binds to both “high-affinity” and “low-affinity” AU-rich regions of specific *yop* transcripts. Intracellular YopD levels decrease as secretion is initiated. 3b. Under medium YopD concentrations the “low-affinity” sites are released and translation proceeds. 3c. Under low YopD concentrations the “high-affinity” sites are also released and translation of these proteins proceeds. This model suggests a hierarchy for *yop*/*ysc* translation that relies on the concentration of YopD in the cell, which in turn is dependent on extracellular conditions.

In *Yersinia* there have been several RNA-binding proteins implicated in the regulation of the T3SS. One of these proteins is YopD, which works in conjunction with YopB to form a pore in the eukaryotic target cell membrane that permits the translocation of T3S effectors into the host cell (Hakansson et al., 1993; Tardy et al., 1999). In addition to its role in translocation, YopD has also been implicated in the negative regulation of the so-called low calcium response (LCR; Williams and Straley, 1998). In *Y. enterocolitica* YopD, together with secretion chaperone LcrH, has been shown to bind *yop* mRNA in the 5′ untranslated region (5′ UTR) of *yopQ* transcripts (known as *yopK* in *Y. pestis*; Anderson et al., 2002). It has been proposed that this binding represses translation from *yopQ* mRNA, perhaps by promoting the degradation of the transcript or by competing with the ribosome for binding (Chen and Anderson, 2011). Furthermore, the half-life of *yopH*, *yopE*, and *yscB* transcripts are longer in a *yopD* deletion mutant of *Y. pestis* compared to wild-type bacteria when grown at 37°C in the presence of calcium, suggesting that the post-transcriptional control of secreted effectors by YopD is not limited to *yopQ/K*. The binding of the YopD-LcrH complex to target transcripts requires two specific AU-rich regions of mRNA that is common to many, but not all *yop* transcripts. The distance of the AU-rich regions from the Shine–Dalgarno site may affect the affinity of YopD for the transcript, suggesting a mechanism for a hierarchy of translation (Figure 1; Chen and Anderson, 2011). Interestingly, these AU-rich regions are not sufficient to confer YopD-LcrH binding to *pla* (plasminogen activator protease) mRNA *in vivo*, nor does mutation of these regions to a more GC-rich content abolish YopD-LcrH binding to *yopH* transcripts *in vitro*. This suggests that the post-transcriptional repression of *yops* is more complex than the effects of these two proteins and the AU-rich regions alone (Chen and Anderson, 2011).

In addition, YscM1 and YscM2 (called LcrQ in *Y. pestis* and *Y. pseudotuberculosis*), which have previously been shown to be negative regulators of the T3SS via a mechanism of feedback inhibition, have also been implicated in the post-transcriptional regulation of *yop* genes in *Y. enterocolitica* (Allaoui et al., 1995; Cambronne and Schneewind, 2002). It has been proposed that the post-transcriptional regulation of the T3SS by the YscM proteins may occur through a mechanism similar to that of YopD-LcrH, and that they may function together with YopD-LcrH to prevent Yop translation (Cambronne and Schneewind, 2002).

Repression of effector Yop translation by YopD-LcrH/YscM1/YscM2 may play a significant role in allowing *Yersinia* to prepare for the translocation of effectors almost immediately upon host cell contact by transcribing low levels of required mRNA, while preventing translation of these effectors before the appropriate time. Although the transcription of *yops* is not strongly activated under non-secretion-inducing conditions, low-level transcription of these genes does occur at 37°C (Cornelis and Wolf-Watz, 1997), which leaves a gap in regulation that may be filled by YopD. During the course of infection *Yersinia* species must carefully balance energy use for growth versus energy use for production of virulence determinants in order to maximize survival and spread, which may explain why this system is so tightly regulated at the transcriptional, post-transcriptional, and post-translational levels. YopD has homologs in nearly every other bacterial pathogen that harbors a T3SS, including *Aeromonas*, *Pseudomonas*, and *Vibrio* species, although no RNA-binding post-transcriptional role for the YopD orthologs has yet been described.

### Csr system

The RNA-binding protein CsrA is the central component of the global carbon storage regulatory system and has been characterized in a number of bacterial pathogens (Altier et al., 2000; Lenz et al., 2005; Heroven et al., 2008; Brencic and Lory, 2009). The Csr system is involved in the regulation of many factors involved in both general metabolism as well as virulence and the response to stress (Timmermans and Van Melderen, 2010). In *Y. pseudotuberculosis*, transcription of *csrA* itself is induced in stationary phase (Heroven et al., 2008). Post-transcriptional regulation by CsrA occurs through the binding of CsrA to nucleotides near the Shine–Dalgarno region of target mRNAs (Liu and Romeo, 1997; Baker et al., 2007). Generally, CsrA binds to GGA-motifs in the 5′ UTR and represses translation by competing with the 30 s ribosomal subunit (Dubey et al., 2003, 2005; Schubert et al., 2007). This also results in accelerated mRNA degradation (Liu et al., 1995). Two highly structured small non-coding regulatory RNA (sRNA) molecules, CsrB and CsrC, control the levels of free CsrA in the bacterial cell, and only CsrA not complexed with CsrB/C can bind to and regulate target mRNA (Liu et al., 1997; Romeo, 1998). In *Yersinia*, *csrB* is transcriptionally regulated by the two-component system BarA/UvrY in response to unknown extracellular signals that likely include nutrient availability, while the mechanism of *csrC* transcriptional regulation in *Yersinia* is not yet known (Heroven et al., 2008). Both CsrB and CsrC possess multiple GGA-motifs that are able to bind CsrA, which then derepresses the post-transcriptional targets of CsrA (Heroven et al., 2008). The sequences of *csrB* and *csrC* in *Y. pseudotuberculosis* are not well conserved with respect to *E. coli* and *Salmonella*, and seem to have a more complex transcriptional regulation than in these species (Heroven et al., 2012).

Microarray analysis of a *csrA* deletion mutant of *Y. pseudotuberculosis* compared with wild-type bacteria showed that CsrA influences, either directly or indirectly, the transcript levels of approximately 500 open reading frames, 3% of which are virulence- or stress-associated (Heroven et al., 2012). Phenotypically, CsrA mutants of *Y. pseudotuberculosis* are aflagellate and non-motile (Heroven et al., 2012). While it is not yet known if a *csrA* mutant in *Yersinia* is attenuated in animal models of infection, the microarray study suggests that *csrA* may regulate host cell invasion, which is a key step in virulence (Heroven et al., 2012). Finally, CsrA regulates the global transcriptional regulator RovA of *Y. pseudotuberculosis*, thereby indirectly controlling the expression of a number of genes, some of which are involved in virulence (Cathelyn et al., 2006; Heroven et al., 2008). With respect to other pathogens, CsrA has been shown to play a role in the regulation of *Legionella pneumophila* type IV secretion (Rasis and Segal, 2009), a key virulence determinant, and likely contributes to the virulence of other bacterial species as well.

### Trans-translation

The small protein SmpB is a unique RNA-binding protein that functions in tandem with the small RNA SsrA to rescue stalled ribosomes from incomplete mRNA transcripts (Komine et al., 1994; Keiler et al., 1996; Karzai et al., 2000). Known as trans-translation, this is a vital process for bacteria as stalled ribosomes will stay on transcripts indefinitely and cannot be recycled for new rounds of protein synthesis, which would thereby result in growth arrest and the eventual death of the cell. The RNA molecule SsrA serves as a substitute message for the ribosome, however without SmpB, SsrA is unable to enter the ribosome to participate in trans-translation (Karzai et al., 1999). SmpB binds to aminoacylated-SsrA in a 1:1 ratio that structurally mimics a tRNA where SmpB acts as the anticodon loop, and this faux tRNA enters into the empty A site of a stalled ribosome (Neubauer et al., 2012). This is followed by a transpeptidation reaction linking the unfinished polypeptide chain to SsrA (Karzai et al., 2000). The errant mRNA is subsequently replaced by SsrA, which allows translation to continue until the ribosome reaches the built-in stop codon (Karzai et al., 2000). This results in the addition of an 11-amino acid residue tag to the end of the incomplete protein that marks it for degradation by intracellular proteases (Karzai et al., 2000). In *E. coli* RNase R is the RNase responsible for degradation of these errant mRNA molecules in an SmpB-SsrA-dependent manner (Richards et al., 2006). Normal (unstalled) ribosomes cannot accommodate SmpB due to the structure of SmpB, thereby ensuring only stalled ribosomes are affected (Neubauer et al., 2012). The C-terminal tail of SmpB is required for binding to the SsrA RNA (Miller et al., 2011). While SmpB plays a key role in ribosome rescue, more recently SmpB has been postulated to contribute to other forms of post-transcriptional regulation, although the mechanism remains elusive (Ansong et al., 2009). In addition to general ribosome recycling, SmpB-SsrA also may play an active role in post-transcriptional gene regulation (Abo et al., 2000; Withey and Friedman, 2002). One such example is seen in *E. coli* in which the binding of LacI to the *lac* operator results in incomplete *lacI* transcripts, which are then acted upon by SmpB-SsrA to ultimately result in the degradation of the transcript, thereby reducing the production of LacI. This is hypothesized to support rapid induction of *lac* operon expression in response to lactose availability (Abo et al., 2000).

Disruption of *ssrA* or *smpB* results in growth defects in many bacterial species under various conditions, including *E. coli*, *Salmonella*, *Neisseria gonorrhoeae*, *Mycobacterium genitalium*, and *M. pneumoniae* (Karzai et al., 2000). In *Y. pseudotuberculosis*, SsrA has been shown to contribute to the pathogenesis in a mouse model of infection (Okan et al., 2006), and similarly, SsrA mutants of *Francisella tularensis* and *Salmonella* are also defective for virulence (Ansong et al., 2009; Svetlanov et al., 2012). *In vitro*, *Y. pseudotuberculosis* lacking SmpB and SsrA is more sensitive to sublethal concentrations of translation-specific antibiotics such as streptomycin and chloramphenicol, and in tissue culture models of infection, this mutant shows a twofold decrease in intracellular survival in macrophages and significantly delayed cytotoxicity toward HeLa cells (Okan et al., 2006). Furthermore, transcription of the T3SS master regulator gene *lcrF/virF* is reduced by 50% in the absence of SmpB-SsrA and is not significantly up-regulated under secretion-inducing conditions (Okan et al., 2006). The exact mechanism by which SsrA regulates LcrF/VirF is unknown, although it has been proposed that SsrA may exert its effects by tagging for degradation the transcript of an unknown transcriptional repressor; hence in an *ssrA*-deficient strain the repressor would accumulate and prevent transcriptional activation of T3SS genes by LcrF. SsrA is also critical for the virulence of *Y. pestis* by both the intranasal and intravenous routes (Okan et al., 2010). Mice inoculated intranasally with *Y. pestis* lacking *ssrA* survived a secondary challenge with fully virulent *Y. pestis*, suggesting that this mutant could serve as a potential vaccine candidate to prevent pneumonic plague (Okan et al., 2010).

### Hfq

The small RNA chaperone protein Hfq is a pleiotropic RNA-binding protein that plays a central role in the post-transcriptional regulation of large numbers of genes in bacteria. This protein is small (101 amino acids in *Yersinia*), and forms a homohexameric ring complex that allows Hfq to bind more than one RNA molecule simultaneously (Kajitani and Ishihama, 1991; Vytvytska et al., 1998; Sauter et al., 2003). Although not ubiquitous, Hfq has been identified in many bacterial species and is known to bind AU-rich regions of RNA (Sun et al., 2002; Zhang et al., 2002; Sobrero and Valverde, 2012). Binding of RNA to Hfq can either stabilize or promote the degradation of mRNA transcripts, depending on the specific interaction (Vytvytska et al., 1998; Masse et al., 2003; Meibom et al., 2009). Furthermore, Hfq also binds to sRNA molecules and enhances the RNA–RNA interaction between the sRNA and its target mRNA (Moller et al., 2002; Zhang et al., 2002; Vecerek et al., 2003). Hfq has been shown to be essential for the virulence of both *Y. pestis* and *Y. pseudotuberculosis* in mouse models of bubonic plague and Yersiniosis, respectively (Geng et al., 2009; Schiano et al., 2010). Although no study has yet been performed to evaluate the effects of an *hfq* deletion in *Y. enterocolitica*, Hfq has been implicated in the negative regulation of the heat stable enterotoxin gene *yst* and thus it is quite likely that Hfq plays a role in the virulence of this pathogen as well (Nakao et al., 1995).

**Figure 2:**
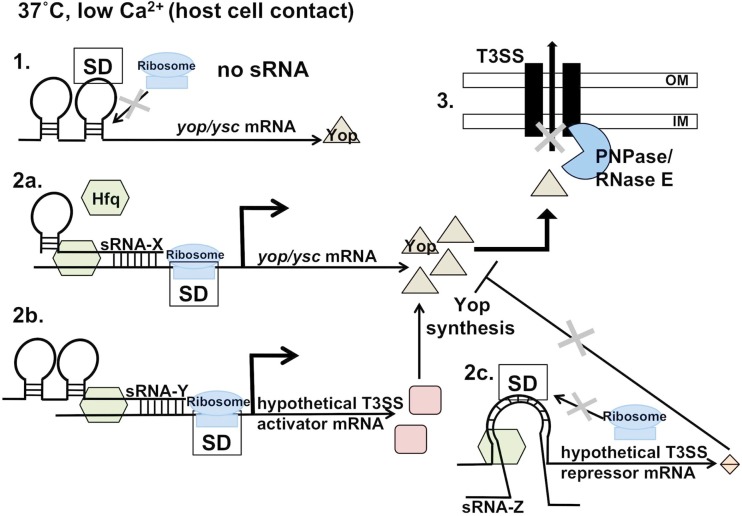
**Hfq-mediated regulation of Yop/Ysc production and ribonuclease-mediated secretion**. Upon host cell contact (or *in vitro* in low calcium concentrations), Yop production, and secretion/translocation occurs. 1. In the absence of one or more hypothetical, Hfq-dependent sRNAs, Yop production occurs at a lower level. 2a. Hfq-dependent sRNA stimulation of translation or stabilization of the transcript may be direct, through binding of the sRNA to the 5′ UTR of the mRNA, leading to increased Yop production. 2b. Alternatively, regulation may be indirect through Hfq-dependent stimulation/stabilization of a secondary post-transcriptional activator of the T3SS, or 2c. Hfq-dependent repression/degradation of a post-transcriptional repressor of T3SS. Both of these scenarios would result in increased Yop production in an sRNA-dependent, post-transcriptional manner. Upon Yop production, PNPase and RNase E may block secretion/translocation of the Yop at a terminal step if conditions are non-ideal.

The pleiotropic nature of Hfq, in that it facilitates many different sRNA-mRNA interactions, has made it difficult to elucidate all of the sRNAs and targets responsible for the Hfq-dependent phenotypes that have been observed so far. The loss of Hfq from *Y. pestis* leads to a significant growth defect when cultured *in vitro* at 37°C, but surprisingly the same growth defect is not observed for *Y. pseudotuberculosis* (Bai et al., 2010; Schiano et al., 2010; Bellows et al., 2012). Growth defects of *hfq* mutants in other bacterial species (*Brucella abortus*, *Vibrio cholerae*, and *Salmonella*) are more similar to the minor defects observed *in vitro* for *Y. pseudotuberculosis*, while more pronounced defects are seen *in vivo* (Robertson and Roop, 1999; Ding et al., 2004; Sittka et al., 2007). Consistent with these data, both *Y. pestis* and *Y. pseudotuberculosis* lacking *hfq* are impaired for intracellular survival in tissue culture cells compared to wild-type, suggesting that one or more Hfq-dependent sRNAs may directly or indirectly regulate critical factors required for intracellular survival (Geng et al., 2009; Schiano et al., 2010). Additionally, there is a significant reduction in T3SS effector protein production in the absence of Hfq in *Y. pseudotuberculosis*, which suggests that one or more sRNAs positively regulate the *Yersinia* T3SS (Figure 2; Schiano et al., 2010). Taken together, these data suggest that *Yersinia* require Hfq to with-stand the stressful conditions found within the mammalian host, although the Hfq-dependent virulence defects in the mammal may be due to general growth deficiencies in addition to the loss of properly functioning virulence determinants. Similarly, Hfq has been implicated in the virulence of other pathogens (Christiansen et al., 2004; Sittka et al., 2007; Kulesus et al., 2008; Fantappie et al., 2009; Meibom et al., 2009; Kendall et al., 2011). For example in *Salmonella*, HilD is positively regulated post-transcriptionally in an Hfq-dependent manner, but it is not yet known if the regulation is indirect through an RNA-binding protein or direct through association with an sRNA (Ellermeier and Slauch, 2007; Sittka et al., 2008). Hfq has been implicated in the regulation of T3SS in other bacteria as well, which suggests that the involvement of Hfq-dependent sRNAs here is a conserved theme in bacterial pathogens (Pfeiffer et al., 2007; Mitobe et al., 2009; Shakhnovich et al., 2009; Kendall et al., 2011).

As with other pathogens, the number of genes regulated through Hfq in *Yersinia* species is likely to be large. Microarray studies on the Hfq-affected transcriptome in *Y. pestis* revealed 243 genes with twofold or greater difference in transcript levels in the absence of *hfq* compared to wild-type bacteria (Geng et al., 2009). Of these, 139 were down-regulated and 104 were up-regulated, and genes belonging to metabolism functional classes are overrepresented in the transcriptome when compared to the genome at large (Geng et al., 2009). This study also found that 23% of these transcripts are related to pathogenicity or stress response processes, including the plasminogen activator protease gene *pla*, the F1 antigen gene *cafI*, the diguanylate cyclase gene *hmsT*, and half of the genes of the T3SS (Geng et al., 2009). Interestingly, the abundance of these T3SS-associated transcripts is elevated in the absence of *hfq*, suggesting that Hfq may be a negative regulator of these genes in *Y. pestis*; this, however, is in contrast to the positive regulation of T3S effector protein levels by Hfq in *Y. pseudotuberculosis* (Geng et al., 2009; Schiano et al., 2010). Hfq-dependent regulation of the T3SS may be divergent between the two species, but a more likely explanation is that in the absence of Hfq, decreased Yop protein production leads to increased transcription of *yop* mRNA through a feedback loop (Allaoui et al., 1995). In addition, there may be other targets of Hfq not identified by this study, as Hfq functions at the post-transcriptional level and thus its absence may not alter the transcript abundance of every Hfq-regulated gene (Koo et al., 2011).

**Figure 3:**
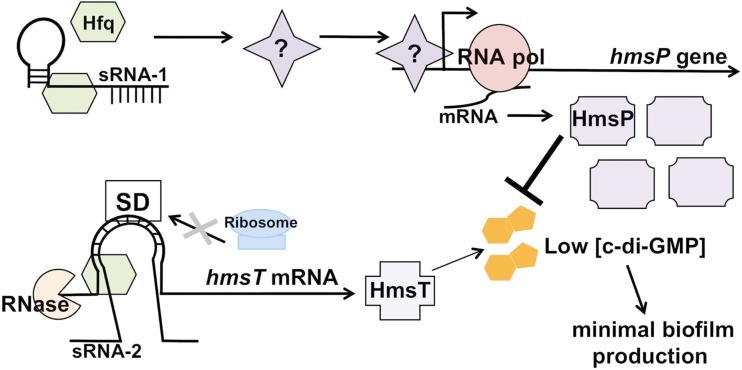
**Hfq-dependent regulation of biofilm formation in *Yersinia pestis***. Hfq, in conjunction with one or more hypothetical sRNAs, is predicted to indirectly affect transcript levels of *hmsP* by either increasing the levels of a transcriptional activator or by decreasing levels of a repressor, thereby stimulating the synthesis of the c-di-GMP phosphodiesterase HmsP. Simultaneously, Hfq (and presumably a cognate *hmsT*-binding sRNA) represses translation of the transcript encoding the diguanylate cyclase HmsT through increasing transcript degradation and possibly blocking translation initiation. These coordinately regulated activities result in low c-di-GMP levels, leading to minimal biofilm production by *Y. pestis*.

Hfq also contributes to other virulence-associated phenotypes in the *Yersiniae*. For instance, Hfq represses the non-flagellar-dependent, swarming motility of *Y. pseudotuberculosis*, as a deletion mutant of *hfq* is hyper-motile on low percentage agar plates (although interestingly this phenomenon is not observed in *Y. pestis*; Schiano et al., 2010). The regulation of this form of motility via Hfq may play a role for *Y. pseudotuberculosis* and perhaps *Y. enterocolitica* during their free-living phase in environment, or may contribute to the cessation of swarming upon entering the mammalian host. Indeed, Hfq has also been implicated in the non-mammalian phase of the *Y. pestis* life cycle. Recently it was shown that Hfq is required for biofilm production in the proventriculus of the flea (Rempe et al., 2012), which suggests that Hfq enables the efficient transmission of *Y. pestis* from the flea vector to the mammalian host (Jarrett et al., 2004). *In vitro*, Hfq represses biofilm formation through the reduction of cyclic diguanylate (c-di-GMP) levels by reciprocally regulating the abundance of both HmsP and HmsT, which are the c-di-GMP phosphodiesterase and major diguanylate cyclase, respectively (Bellows et al., 2012). Hfq contributes to the regulation of HmsP at the transcriptional level, while HmsT is directly regulated via Hfq at the post-transcriptional level (Figure 3; Bellows et al., 2012). These coordinate but distinct regulatory processes may allow *Y. pestis* to link the post-transcriptional control of biofilm formation to environmental sensing, although it is not yet known whether this occurs in the flea, the mammal, or both. Hfq has also been implicated in the regulation of biofilms produced by uropathogenic *E. coli* and *V. cholerae*, suggesting that the contribution of Hfq to biofilm formation, though perhaps not the mechanism, is a conserved one in the bacterial world (Kulesus et al., 2008; Bardill et al., 2011).

## Non-Coding RNAs

Non-coding RNAs encompass a large and diverse group of RNA molecules that do not result in the translation of a protein product. Instead, many are used in regulatory roles or other functional capacities upon transcription.

### *Cis-* and *trans*-acting sRNAs

A major class of non-coding RNAs includes the *trans*-acting sRNAs, which by definition are encoded at genomic loci distal to those of their targets. These small RNAs have recently become appreciated for their roles in the post-transcriptional regulation of many bacterial genes, including virulence-associated genes in both *Yersinia* and other bacterial pathogens. In general, *trans*-acting sRNAs range in size from 50 to 500 nt in length, are highly structured, and usually base pair with target mRNA within the 5′ UTR to either repress or stimulate translation (Gottesman, 2005). The ability of the sRNA to base pair with its target is the basis for this type of regulation, although this base pairing is usually imperfect over the span of 7–10 nt (the “seed” sequence; Gottesman, 2005). Additionally, *trans*-acting sRNAs often require the chaperone protein Hfq for their expression, stability, and/or function. Hfq is thought to simultaneously bind to both the sRNA and mRNA, and in so doing facilitate the interaction of sRNAs with their targets. Alternately, *cis*-acting sRNAs generally do not require Hfq for base pairing as their genomic locations, usually antisense to their target mRNAs, allow for extensive Watson–Crick interactions between the sRNA and target (Gottesman and Storz, 2011). Several publications identifying various cohorts of non-coding sRNAs expressed by *Yersiniae* have recently been published. Using a deep sequencing-based approach, Koo et al. (2011) identified 150 previously unannotated sRNAs in *Y. pseudotuberculosis* (termed Ysrs for *Yersinia* small RNAs), and Qu et al. (2012) used cDNA cloning for the identification of 43 sRNAs in *Y. pestis*, 25 of which are predicted to be *cis*-acting. While the functions of most of these sRNAs have not yet been elucidated, those that have and other specific examples are discussed here.

### sRNAs/Ysrs that target the outer membrane

The synthesis of outer membrane proteins is often highly regulated at the post-transcriptional level. RybB is a well conserved Hfq-binding sRNA in the enterobacteriaceae that has been shown in both *E. coli* and *Salmonella* to down-regulate the production of multiple outer membrane proteins including OmpA, OmpC, OmpD, OmpF, OmpW, and others by promoting accelerated mRNA degradation (Johansen et al., 2006; Papenfort et al., 2006). This sRNA (also known as Ysr48 in *Yersinia*) is present in all three pathogenic *Yersinia* species, but does not seem to play a major role in the pathogenesis of either *Y. pestis* or *Y. pseudotuberculosis* in mouse models of infection (although a more subtle role for RybB/Ysr48 in *Yersinia* virulence cannot not be ruled out; Koo et al., 2011). While the targets of RybB/Ysr48 in *Yersiniae* have not yet been identified, as many of the RybB-regulated genes in other species are conserved in *Yersinia* there is likely to be some overlap.

MicF is another highly conserved sRNA of the enterobacteriaceae that regulates the outer membrane protein OmpF in *Yersinia* species. The mechanism of MicF regulation is well understood in *E. coli*, with an approximately 20 base pair imperfect RNA duplex that forms within the Shine–Dalgarno region of the *ompF* mRNA (Schmidt et al., 1995). This base pairing prevents translation of OmpF and may lead to the degradation of the message (Andersen et al., 1989; Delihas and Forst, 2001). The binding of MicF to *ompF* in *Yersinia* is predicted to be very similar, with the same expected outcome of repression by translation inhibition and degradation of the *ompF* transcript (Delihas, 2003). Two other conserved sRNAs, MicA/Ysr7, and OmrA/Ysr149 that (based on their homologies with other species) are also predicted to target mRNAs encoding conserved outer membrane proteins in *Yersinia* (Udekwu et al., 2005; Guillier and Gottesman, 2006; Vogel and Papenfort, 2006). These sRNAs are the most abundantly expressed Ysrs during the *in* *vitro* growth of *Y. pseudotuberculosis* at 37°C (Koo et al., 2011), although their contributions to the biology of the *Yersiniae* have not yet been established.

### GcvB/Ysr45

The conserved sRNA gene *gcvB*/Ysr45 encodes not one but two sRNAs in *Yersinia*, and these sRNAs have been shown to be repressors of *dppA*, which encodes the periplasmic-binding protein component of the dipeptide transport system in *Y. pestis* (McArthur et al., 2006). The two GcvB molecules have different termination sites and are 130 and 206 nt in length, and in *E. coli* the 206-nt form is required for repression of *dppA*; whether this is conserved in *Yersinia* remains to be determined (Urbanowski et al., 2000; McArthur et al., 2006). As the regulation of *dppA* by GcvB occurs post-transcriptionally in *E. coli* (Pulvermacher et al., 2008, 2009), it is likely that the regulation of *dppA* in *Yersinia* is similar. Regulation of the dipeptide transport system in this way could play a role in the *Yersinia* adaptation to stress, as in *E. coli* the modulation of *dppA* occurs in response to environmental signals (Olson et al., 1991). A *gcvB*/Ysr45 deletion in *Y. pestis* results in changes both in colony morphology and generation time, suggesting that GcvB/Ysr45 may regulate multiple targets directly or indirectly (McArthur et al., 2006). As opposed to the high levels of the MicA/Ysr7 and OmrA/Ysr149 sRNAs at 37°C, when *Y. pseudotuberculosis* is cultured *in vitro* at lower temperatures (26°C), GcvB/Ysr45 is the most highly up-regulated *Yersinia* Ysr, although the implications of this are as yet unknown (Koo et al., 2011).

### GlmY/Ysr147 and GlmZ/Ysr148

In *Y. pseudotuberculosis* there exists an interesting sRNA-target relationship between *glmS*, which encodes glucosamine-6-phosphate, a key enzyme in the cell wall synthesis pathway, and the sRNAs GlmY/Ysr147 and GlmZ/Ysr148 (Gopel et al., 2011). GlmZ/Ysr148 stabilizes the *glmS* transcript and therefore acts as a positive post-transcriptional regulator of cell wall synthesis, while GlmY/Ysr147 regulates the levels of GlmZ/Ysr148 through an unknown mechanism (Kalamorz et al., 2007; Reichenbach et al., 2008). This method of *glmS* regulation by GlmZ is highly conserved among the enterobacteriaceae (Urban et al., 2007). Based on the sheer number of targets and the evolutionary conservation of sRNAs that are involved in the regulation of outer membrane proteins, it is clear that the rapid adjustment of the outer membrane is a priority for bacterial species, and that post-transcriptional regulation mediated by these sRNAs is a major mechanism by which to accomplish such regulation.

### YenS

In *Y. enterocolitica*, the sRNA gene *yenS* encodes two small, non-translated RNAs of 165 and 105 nt that have different termination sites but share the same 5′ end (Tsai and Winans, 2011). These two sRNAs were found to inhibit translation and promote degradation of the *yenI* mRNA. YenI is homologous to the autoinducer synthetase LuxI of *Vibrio fischeri* and produces the quorum sensing molecule 3-oxohexanoylhomoserine lactone (OHHL), which at high levels inhibits swarming motility (Shadel et al., 1990; Tsai and Winans, 2011). Thus, the YenS sRNA is an indirect positive regulator of motility in *Y. enterocolitica* that acts through the modulation of YenI production. Transcription of *yenS* is activated in response to low levels of OHHL through YenR, which is homologous to the response regulator LuxR in *V. fischeri* (Dunlap and Ray, 1989). Interestingly, in a *yenI* null strain that is hyper-motile, a second deletion in *yenS* suppresses the hyper-motility phenotype, suggesting a potential secondary role for YenS in the regulation of motility in *Y. enterocolitica* (Tsai and Winans, 2011). A similar non-coding RNA was found in the plant pathogen *Pantoea stewartii* subsp. *stewartii* that is regulated by the LuxR homolog in an OHHL-dependent way, suggesting a conservation of sRNA regulation by YenS in quorum sensing (Schu et al., 2009). While the genomes of *Y. pestis* and *Y. pseudotuberculosis* also encode homologs of the *yenS* gene, this sRNA was not identified during a global screen for sRNAs in *Y. pseudotuberculosis*; it is possible that YenS may not be expressed under the conditions examined in the study or did not meet the filtering/threshold criteria to be identified in the survey (Koo et al., 2011; Tsai and Winans, 2011). Post-transcriptional regulation of quorum sensing may be a common theme among bacterial pathogens and symbionts, as small non-coding RNAs have also been implicated in the regulation of quorum sensing in *V. harveyi* and *V. cholera* (albeit by a seemingly different mechanism; Lenz et al., 2004; Svenningsen et al., 2009; Tu et al., 2010).

### RhyB/Ysr48

Iron acquisition is a key concern for bacterial pathogens; therefore it is not surprising that the enterobacteriaceae possess sRNAs that participate in the regulation of genes involved in such systems (Masse and Gottesman, 2002; Nairz et al., 2010). One such sRNA is RhyB, an Hfq-dependent, Fur-repressed sRNA responsible for the post-transcriptional regulation of the *sdhCDAB* operon and five other genes in *E. coli* and *V. cholerae* (Masse and Gottesman, 2002; Davis et al., 2005). The products of these genes are all iron-storing or iron-using and under low iron conditions, RyhB acts as a negative regulator to repress their translation. Two copies of this sRNA gene, RhyB1/Ysr48.1 and RhyB2/Ysr48.2, are found in *Yersinia* species, and both are thought to function similarly to the canonical RhyB of *E. coli*. Deng et al. (2012) recently showed that although RhyB1/Ysr48.1 requires Hfq for its stability, RhyB2/Ysr48.2 does not, and that the expression of both sRNAs is highly up-regulated in the lungs of mice infected with *Y. pestis*, known to be an iron-limiting environment. However, deletions of these sRNAs do not result in decreased bacterial burden in the lungs of infected mice, suggesting that they may not play a vital role in iron acquisition by *Y. pestis* during pneumonic plague. It remains unclear as to why *Y. pestis* carries two copies of RhyB/Ysr48 and if there is a biological consequence for this gene duplication during infection of either the mammal or the flea.

### SgrS/Ysr150

An unusual sRNA conserved among enterobacteriaceae is SgrS (Horler and Vanderpool, 2009). This sRNA not only functions in the traditional sense by base-paring with a target mRNA to regulate translation, but in many bacteria is itself also translated into a small 43 amino acid protein known as SgrT (Wadler and Vanderpool, 2007). SgrS is induced under glucose-phosphate stress conditions and utilizes traditional Hfq-dependent base pairing within the 5′ UTR to negatively regulate *ptsG* [EIICB^Glc^ of the phosphoenolpyruvate phosphotransferase system (PTS)] post-transcriptionally, which in *E. coli* prevents the synthesis of new glucose transporters. SgrT helps to rescue the bacterial cell from glucose-phosphate stress by inhibiting glucose transporter activity at the post-translational level (Wadler and Vanderpool, 2007). Recent evidence suggests that SgrS may also regulate other PTS genes (Rice and Vanderpool, 2011). The base-paring function of SgrS/Ysr150 with *ptsG* is conserved in *Y. pestis*, however the sRNA does not have the same 5′ end as the *E. coli* *sgrS*, and therefore does not produce SgrT (Wadler and Vanderpool, 2009). The lack of SgrT in *Y. pestis* may alter the bacterial response to glucose-phosphate stress compared to other organisms, however there could also be an unknown redundant system that *Y. pestis* utilizes instead.

### SraG

One conserved sRNA present in *Yersinia* with no assigned function in other enterobacteriaceae to date is SraG (Argaman et al., 2001; Sridhar et al., 2009). An attempt to determine targets of SraG in *Y. pseudotuberculosis* using a proteomic screen followed by genetic analyses identified the operon YPK_1206-05 as a potential target. The authors of this study conclude that SraG negatively regulates this operon directly at the post-transcriptional level (Lu et al., 2012). This operon has not been annotated, but secondary structure prediction suggests that it YPK_1206 is similar to an IHF-like DNA bending protein.

### Ysr29 and Ysr35

Koo et al. (2011) detected almost all of the conserved sRNAs discussed above in a global analysis of non-coding RNAs expressed by *Y. pseudotuberculosis*, as well as an additional 118 previously unidentified, putative sRNAs that are specific for *Y. pseudotuberculosis* and/or *Y. pestis*, but absent from other enterobacterial species. In an effort to provide a consistent naming convention for these sRNAs across all *Yersinia* species, going forward the authors propose a standard nomenclature for newly identified non-coding RNAs in *Yersiniae* that adopts the “Ysr” naming convention. Twenty-nine of the Ysrs identified by Koo et al. (2011) were detected by northern blot analysis, and one *Y. pseudotuberculosis*-specific sRNA, Ysr29, was found to contribute significantly to mortality in a mouse model of Yersiniosis. By using 2D differential gel electrophoresis (DIGE) combined with mass spectrometry, eight genes were identified as potential regulated targets of Ysr29: *ureC*, *ahpC*, *gst*, *frr*, *rpsA*, *ompA*, *groEL*, and *dnaK* (Koo et al., 2011). None of these targets showed a significant change in transcript level in the absence of *ysr29*, suggesting that Ysr29 affects protein levels through post-transcriptional mechanisms of regulation. Each of these targets may contribute to the bacterial response to environmental or host stresses, demonstrating that Ysr29 is a major post-transcriptional regulator of the *Y. pseudotuberculosis* adaptation to stress (Koo et al., 2011). A second *Yersinia*-specific sRNA, Ysr35, was also examined for its contribution to virulence. Deletion of this conserved sRNA from the genomes of both *Y. pseudotuberculosis* and *Y. pestis* resulted in decreased virulence in mouse models of Yersiniosis and pneumonic plague, respectively, although it is not yet known if the Ysr35-regulated target(s) are also conserved between the species or if Ysr35 has adapted to specific and/or unique targets between the two (Koo et al., 2011). It is expected that future sRNA identification studies will reveal additional Ysrs not found here, and it will be of particular interest to determine the sRNA-omes of the *Yersiniae* during mammalian infection, flea colonization, and in environmental reservoirs.

### Transcript antisense to *pla*

While the majority of non-coding sRNAs identified in *Yersinia* so far appear to be *trans*-encoded, a number of putative *cis*-acting sRNAs have also been identified. One of these transcripts is encoded on pPCP1, a plasmid specific to *Y. pestis* that carries the genes for pesticin, the pesticin immunity protein, and the plasminogen activator protease Pla (Sodeinde and Goguen, 1989). Pla is an essential virulence factor required by *Y. pestis* during both bubonic and pneumonic plague (Sodeinde et al., 1992; Lathem et al., 2007). Sodeinde and Goguen (1989) identified a transcript complementary to the coding sequence and upstream region of *pla* that may be expressed from a promoter with high similarity to the *E. coli* sigma70 -10 and -35 sites. While a potential 48-amino acid polypeptide may be encoded within this sequence, the authors were unable to detect a protein produced from the transcript. Instead, this antisense molecule may participate in the regulation of Pla synthesis, although no physiological role for the anti-*pla* transcript has been determined to date.

### Yp-sR3, Yp-sR7, and Yp-sR8

Among the sRNAs identified by Qu et al. (2012) 25 are predicted to be *cis*-acting. Two of these *cis*-encoded sRNAs in *Y. pestis* strain 201 are Yp-sR3 and Yp-sR8, both of which are maximally expressed during stationary phase *in vitro* (Qu et al., 2012). Yp-sR3 spans a potential operon formed by YP_1329 and YP_1330 that encodes two putative membrane proteins (Qu et al., 2012). This suggests that Yp-sR3 could also participate in the regulation of the *Yersinia* membrane through an antisense mechanism. A third *cis*-encoded RNA found is Yp-sR7, which is transcribed from the strand opposite the gene *rplK* that encodes the 50S ribosomal protein (Qu et al., 2012), but its function is as yet unknown. Indeed, much work remains to be done to determine the contributions of these and other *cis*-acting sRNAs to the biology of the *Yersiniae*.

## RNases

RNases cleave RNA transcripts internally (endonucleases) or from the 5′ or 3′ end (exonucleases). The average half-life of mRNA transcripts in *E. coli* ranges from 1 to 10 min, which allows the bacterial cell to respond quickly to various surroundings, and RNA degradation is mediated by a degradasome that includes the endonuclease RNase E (Feng and Niu, 2007). The breakdown of RNA transcripts by the degradasome is essential to normal bacterial cell metabolism, but targeted degradation of mRNA also plays an important role in the post-transcriptional regulation of gene expression. For example, binding of the sRNA RyhB to mRNA targets in the presence of Hfq results in RNase E-dependent degradation of the sRNA::transcript complex (Masse et al., 2003). Hfq associates with RNase E through the C-terminal scaffold of RNase E, which is thought to be a general mechanism for the repression of protein production through sRNAs (Aiba, 2007).

As in other Gram-negative bacteria, RNase E is conserved in *Yersinia* species. While it has not yet been possible to generate an *rne* mutant of *Yersinia*, *Y. pseudotuberculosis* carrying a dominant negative version of the gene for RNase E that lacks the C-terminal scaffolding domain is defective for survival within macrophage-like cells (Yang et al., 2008). RNase E has also been implicated in the regulation of the T3SS, as the dominant negative-containing mutant also showed decreased secretion of YopE into culture supernatant. Interestingly, the amount of YopE isolated from cell lysates is unchanged, suggesting that RNase E regulates the T3SS at a post-translational step and not by the degradation of T3SS effector mRNA transcripts at the post-transcriptional level (Yang et al., 2008). A second exonuclease known as polynucleotide phosphorylase (PNPase) also contributes to the regulation of the T3SS in *Yersinia* species in a manner that is predicted to function at the same level as RNase E (Figure 2; Rosenzweig et al., 2005, 2007). The S1 RNA-binding domain of PNPase is required for the optimal export of effectors by the T3SS, but is independent of the ribonuclease activity of PNPase, as the catalytic activity of PNPase is required for growth of *Yersinia* at low temperature (Goverde et al., 1998; Rosenzweig et al., 2005; Rosenzweig and Schesser, 2007). PNPase is also associated with the regulation of the T3SS of *S. enterica*, however in this case the regulation is indirect and occurs at the transcriptional level (Clements et al., 2002). There are numerous other RNases that play critical roles in the post-transcriptional regulation of virulence genes, and although many of these RNases are conserved in *Yersinia*, the mechanisms of action and the targets involved have not been elucidated (Arraiano et al., 2010).

## Thermoswitches/Thermosensors

The regulation of virulence in *Yersinia* is largely dependent on the ability of the bacteria to sense the temperature of their surroundings, and as such a significant number of genes are thermally regulated. Whole genome DNA microarray analysis of *Y. pestis*, for example, revealed that over 400 genes are differentially regulated at 26 and 37°C, with 39% induced and 61% repressed between the two temperatures (Han et al., 2004). A number of these genes include those encoding the T3SS. The T3SS is activated upon transition from the environmental niche to the mammalian host by the de-repression of transcription of *lcrF* (also known as *virF*), a gene encoding an AraC-like transcriptional activator of the T3SS. LcrF globally up-regulates the transcription of the genes for both the secretion apparatus (*yscs*)and effectors (*yops*). It has long been known that the transcription of *lcrF* is thermoregulated, but new data demonstrate that, as predicted by Hoe and Goguen (1993), the translation of LcrF is also controlled in a temperature-dependent manner (Hoe and Goguen, 1993; Bohme et al., 2012). Translation of LcrF is modulated through a secondary structure within the intergenic region of the *yscW-lcrF* transcript that traps the Shine–Dalgarno sequence of *lcrF* at low temperatures and thus prevents translation (Figure 1). This secondary structure forms a motif that is also observed for heat-shock genes in many eubacteria (Kortmann and Narberhaus, 2012; Schumann, 2012), and in *Yersinia* the *lcrF* thermosensor consists of a two-stem loop structure that forms at 25°C (Bohme et al., 2012). At 37°C, however, the stem-loops denature, the Shine–Dalgarno site becomes accessible to the ribosome, and translation is initiated (Bohme et al., 2012). The authors of this study found that if the *lcrF* thermosensor is mutated to create a permanently open or permanently closed conformation, the virulence of *Y. pseudotuberculosis* is reduced in a mouse model of Yersiniosis, which suggests that the ability to fine tune the translation of LcrF – either up or down – is critical to the virulence of this pathogen. So far only one other virulence-associated thermosensor has been identified in a bacterial pathogen: the *prfA* gene (a positive regulator of listeriolysin) of *Listeria monocytogenes* (Johansson et al., 2002; Loh et al., 2012). Given the major contribution of temperature to the biology of *Yersinia*, it is possible that there may be other thermosensors that remain unidentified, including those that regulate virulence genes.

## Riboswitches

One mechanism of post-transcriptional regulation that has not been well studied in *Yersinia* is the use of riboswitches to control gene expression. A riboswitch is similar to the thermosensor/thermoswitch of LcrF discussed above in that it is usually (but not always) *cis*-encoded within the 5′ UTR of the regulated mRNA and forms a secondary structure that prevents translation under certain conditions (Mandal and Breaker, 2004). Where a riboswitch differs, however, is that the relief of this secondary structure depends on the binding of a ligand rather than changes in temperature, and in response to the presence or absence of the ligand a riboswitch may influence mRNA processing or transcript termination in addition to translation initiation. This ligand is often a metabolite that serves as a sensor of available nutrients (Mandal and Breaker, 2004). For example, in *V. cholerae* a glycine-binding riboswitch upstream of the *gcvT* operon acts as an on-switch in the presence of glycine to increase production of proteins that form the glycine cleavage system (Mandal et al., 2004). While many riboswitches contribute to the regulation of general metabolism or cellular homeostasis, some have been shown to participate in virulence factor expression. For instance, in *L. monocytogenes* the *trans*-acting *S*-adenosylmethionine (SAM)-riboswitch SreA is activated in the presence of SAM, which is then able to bind to and repress PrfA translation at 37°C, thereby linking a metabolite-sensing riboswitch directly to virulence (Loh et al., 2009; Xayarath and Freitag, 2009). As translation of PrfA is also regulated by a thermosensor, the dual post-transcriptional regulation of *pfrA* by secondary RNA structures demonstrates the need for tight regulation of the *pfrA* regulon in *L. monocytogenes*.

Conserved riboswitches in *Yersinia* species have been found through bioinformatic analyses. The molybdenum cofactor (Moco)-sensing riboswitch, which controls expression of adjacent genes in response to the presence of Moco, is conserved in *Y. pseudotuberculosis* (Regulski et al., 2008). Additionally, the co-enzyme B_12_ riboswitch, which is responsible for the translational repression of cobalamin-transport protein production at high concentrations of the co-enzyme, is also conserved in *Y. pestis* (Nahvi et al., 2004). Neither of these conserved riboswitches have been experimentally validated in *Yersinia*. One riboswitch that has been recently experimentally tested in *Y. enterocolitica*, however, is found within the 5′ UTR of the gene *mgtA*, which responds to Mg^2+^ concentrations to regulate expression of the magnesium transporter protein (Korth and Sigel, 2012). In *S. enterica* high Mg^2+^ concentrations lead to the early Rho-dependent termination of *mgtA* transcription through a conformational change in the RNA, thereby regulating magnesium transporter production (Hollands et al., 2012). Given the widespread and integral nature of many riboswitches (Winkler and Breaker, 2005)it is likely that *Yersinia* may encode additional riboswitches.

## Conclusion

Post-transcriptional regulation in *Yersinia* occurs through multiple different mechanisms, many of which have been shown enhance the survival and virulence of these species. The studies performed so far on post-transcriptional regulation in *Yersinia* have focused heavily on the T3SS, which is reasonable given the central role of the T3SS to pathogenesis. While the complexity of the T3SS in *Yersinia* is great, further investigation into the post-transcriptional mechanisms that govern the optimal expression of the T3SS, including the identification of additional sRNAs, thermosensors, and the like, may fill in some of the gaps that remain after analysis of transcriptional regulation of the T3SS is exhausted.

The number of putative sRNAs identified by both deep sequencing and cDNA cloning of the *Yersinia* transcriptome have provided many avenues for the study of post-transcriptional mechanisms of gene regulation in these species (Koo et al., 2011; Qu et al., 2012). Although many sRNAs are conserved between related bacterial species, of the Ysrs identified in *Y. pseudotuberculosis* by Koo et al. (2011) 79% were found to be *Yersinia-*specific and not present in other genera such as *Escherichia* or *Salmonella*. Additionally, 56% of the *Yersinia-*specific sRNAs have nucleotide mismatches or other sequence differences between *Y. pseudotuberculosis* and *Y. pestis* (Koo and Lathem, 2012). These disparities, though small, may be significant as a single mismatch in the RNA–RNA-binding site of an sRNA-mRNA pair can abolish function of the sRNA (Hao et al., 2011). Furthermore, Koo et al. (2011) found that many sRNAs with identical sequences were differentially expressed between the two species with respect to growth phase, temperature and requirement for Hfq. These differences should not be overlooked when examining the mechanisms of gene regulation between the species and may inform further studies on the evolution of *Yersinia* as a human pathogen (Lathem, 2012). The variation between the sRNA-omes of *Y. pestis*, *Y. pseudotuberculosis*, and *Y. enterocolitica*, both *in vitro* and *in vivo*, may hold clues that help explain the stark differences between the disease etiologies of the enteric *Yersinia* and the plague bacillus that are not made clear by analyses of simple virulence gene acquisitions or losses.

The mechanisms detailed in this review emphasize the importance of post-transcriptional regulation in *Yersinia* species. Rapid adaptation to changing conditions through the action of RNA-binding proteins and several classes of non-coding RNAs are key for survival and propagation of these bacteria. In particular, post-transcriptional regulation plays an integral role in virulence, and not just in the control of metabolic pathways. The variety of post-transcriptional mechanisms in *Yersinia* makes it an excellent model organism for studying this growing field of regulation.

## Conflict of Interest Statement

The authors declare that the research was conducted in the absence of any commercial or financial relationships that could be construed as a potential conflict of interest.
